# Assessment of sociodemographic and psychiatric characteristics of transgender adults seen at a Midwest tertiary medical center

**DOI:** 10.3389/fendo.2024.1445679

**Published:** 2024-09-04

**Authors:** Samuel Cortez, Dominic Moog, Elizabeth Baranski, Kelley Williams, Jinli Wang, Ginger Nicol, Thomas Baranski, Cynthia J. Herrick

**Affiliations:** ^1^ Department of Pediatrics, Division of Endocrinology and Diabetes, Washington University School of Medicine, Saint Louis, MO, United States; ^2^ Washington University School of Medicine, Saint Louis, MO, United States; ^3^ Department of Medicine, Division of Endocrinology, Diabetes, and Lipid Research, Washington University School of Medicine, Saint Louis, MO, United States; ^4^ Center for Biostatistics and Data Science, Washington University School of Medicine, Saint Louis, MO, United States; ^5^ Department of Psychiatry, Washington University School of Medicine, Saint Louis, MO, United States; ^6^ Department of Surgery, Division of Public Health Sciences, Washington University School of Medicine, Saint. Louis, MO, United States

**Keywords:** transgender, socioeconomical impact, mental health, gender affirming health care, retrospective study

## Abstract

**Background:**

The National Academy of Medicine has formally identified transgender adults as an understudied population in critical need of health research. While national surveys, like the US Transgender survey, have characterized higher rates of depression, anxiety, suicidality and socioeconomic need in the transgender community, studies have not examined the impact of sociodemographic and clinical characteristics on mental health related outcomes.

**Objective:**

To describe the sociodemographic and mental health characteristics of transgender adults seen at a large Midwest transgender clinic and to determine factors associated with self-reported mental health conditions.

**Methods:**

Descriptive, retrospective, cross-sectional study of new transgender patients 18 years and older seen at a large Midwest transgender clinic between December 2019 and June 2022.

**Results:**

A total of 482 charts were reviewed. During their initial evaluation, 11.6% (56/482) reported having a history of suicide attempt and 81.3% (392/482) reported a mental health diagnosis with the most common being depression, anxiety, attention deficit disorder, and post-traumatic stress disorder. Multivariable logistic regression results show no single factor was significantly associated with mental health diagnosis after adjusting for the effect of age and race. Patients who were new to gender affirming hormone therapy (54%, 254/468) are 2.0 (95% CI 1.4-2.9) times more likely to report having a mental health care provider than patients who were seen for continuation of therapy (46%, 214/468). Ten records with race not disclosed, 3 records with gender identity “other” and 2 records with gender identity not disclosed were excluded from analysis.

**Conclusion:**

This study reinforces the finding that transgender adults have an increased lifetime prevalence of mental health conditions. The higher prevalence of mental health conditions in our clinic was not associated with sociodemographic factors included in the study. Furthermore, transgender patients are less likely to have seen mental healthcare providers after initiation of gender affirming hormone therapy.

## Introduction

Transgender individuals experience discordance between their gender identity and their sex assigned to them at birth (1–3). Some transgender individuals experience gender dysphoria (GD), which the Diagnostic and Statistical Manual of Mental Disorders (DSM-5) defines as “clinically significant distress or impairment in social, occupational, or other key areas of functioning.” GD affects psychosocial development and has been associated with psychiatric conditions like depression and suicidal ideation (4–6).

Despite this knowledge and having a large body of literature characterizing psychiatric diagnosis in various subsets of the U.S. cisgender population, there is a paucity of data quantifying the prevalence of psychiatric diagnosis and its burden in the adult transgender population (7).

Studies using self-reports of gender identity and its variants suggest that 0.17% to 1.3% of young adults identify as transgender (8). Even using the conservative estimate of 0.3%, the number of people living in the United States who identify as transgender is nearly 1 million (9).

The National Academy of Medicine has identified transgender adults as an understudied population in critical need of health research (10). Research in transgender adults is critical because they are particularly vulnerable to the impact of these stressors including governmental policies limiting participation in sports or access to gender affirming hormone therapy (GAHT) (11), employment discrimination leading to lack of health insurance, and refusal of health care services which would affect their socioeconomic outcomes (12–14). Socioeconomic status is associated with a number of health related outcomes (15), and it is important to understand the impact of socioeconomic status on the mental health outcomes in transgender individuals (16, 17).

Our study aims to address this knowledge gap by describing the sociodemographic and mental health characteristics of a sample of transgender adults establishing care at a Midwest tertiary medical center transgender clinic. We also describe factors associated with general mental health, including self- report of a mental health diagnosis, being prescribed psychotropic medications, and/or being seen by a licensed behavioral health professional.

## Methods

This is a descriptive, retrospective, cross-sectional study. Participants included transgender patients 18 years and older seen at initial visits at the Washington University Adult Transgender Center in St. Louis, MO between December 2019 and June 2022. Patients were classified in two groups: (1) new to GAHT and (2) those continuing GAHT. The adult transgender center follows the informed consent model in which patients are placed at the center of the decision-making process (18).

While longitudinal data were collected, this analysis reports cross-sectional data at baseline. The Institutional Review Board of Washington University in St. Louis approved this research; IRB ID 202202153 in February of 2022.

### Study procedures

All data were extracted from the electronic health record (Epic) into a de-identified Research Electronic Data Capture (REDCap) database. The population of interest was identified using Slicer Dicer to select patients 18 years of age and older having “transgender new” as visit type and department of endocrinology as department between December 1, 2019, and June 30, 2022. Two members of the study team extracted all data (EB, DM). Additional study team members (DM, SC) performed quality assurance on 10% of collected data by verifying collected data against medical record. A separate key linking study identifier to medical record number and protected health information was kept in a password protected file on a secure server. Data extracted from the medical record at each participant’s initial visit included:

1. Demographic data: age, race, ethnicity, sex assigned at birth, gender identity, primary and secondary health insurance, and Zip code of residency to obtain area of deprivation index.
2. Psychiatric history: Self-reported mental health diagnoses, mental health history including suicidal attempts, mental health providers defined as psychiatrist or therapist and seen over the last year, psychotropic medications prescribed, previous mental health admissions, community referrals provided in clinic.
3. Medical history: Self-reported medical diagnoses, primary care provider, referral from the pediatric center, gender-affirming treatment regimens, interest in and referrals made for gender-affirming surgery, and use of tobacco/nicotine products. The variable assessing whether an individual was referred from a pediatric transgender care provider in our health system is important because comprehensive, multidisciplinary psychosocial evaluation including mental health evaluation was required for all individuals in the pediatric center, per WPATH guidelines for adolescent transgender care. Individuals presenting for care for the first time as adults were evaluated through the informed consent model and were not required to have seen a mental health professional, also per WPATH guidelines.

Information on health insurance was collected because the health care system in the US can be defined as a mixed system of public payers (insurance plans that are funded by federal or state government for individuals with limited financial resources or high health care needs) (19), private insurance, and individual payments (self-pay). Studies have found positive and significant effects of health insurance coverage on a range of health outcomes (20–23). We further divided those with health insurance coverage between those with primary and secondary health insurance. Primary health insurance is the first to pay for medical-related expenses. Secondary health insurance covers all or part of the expenses not covered by the primary health insurance plan; for example, some private insurers offer supplementary plans to cover out-of-pocket costs like deductibles and co-pays that are not covered by the primary insurer.

The area of deprivation index (ADI) was obtained through the neighborhood atlas organized by the University of Wisconsin using U.S. Census Bureau geographies. ADI is associated with an individual’s block group or neighborhood and measures poverty, education, housing, and employment status to provide a factor-based index used as a proxy measure of socioeconomic deprivation (24, 25). The national percentile divides ADI score into 100 equal sections, categorizing the individual block neighborhood, with those in the first percentile being the least disadvantaged and those in the hundredth percentile being the most disadvantaged.

### Statistical analysis

Data are presented as median and interquartile range for continuous data that was not normally distributed, and frequency with percentage for categorical data. The association between gender identities and categorical factors were examined using Chi-square or Fisher’s exact test as appropriate. Primary outcomes of interest were defined dichotomously as 1) reporting a mental health diagnosis and 2) reporting a visit to a mental health provider over the last year. Multivariable logistic regression models were performed to assess the association between sociodemographic and clinical factors and these primary outcomes with age and race as covariates. Odds ratio (OR) values and their respective confidence interval (CI) were estimated. Ten records where race was not disclosed, 3 records where gender identity was reported as “other” with no further information and 2 records where gender identity was not disclosed were excluded from multivariable logistic regression analysis. All analyses were performed using SAS version 9.4 (SAS Institute Inc, Cary, NC). A p-value of less than or equal to 0.05 was used to determine statistical significance.

## Results

A total of 482 charts were reviewed during the study period. The sample had a median age of 24 years and ranged from 18 years to 75 years of age with 46% identifying as transgender women, 38% as transgender male, and 16% as non-binary. Individuals were predominantly white, non-Hispanic (n=433, 89.8%). More than 92.7% had primary health insurance; 17.9% had Medicare or Medicaid versus 72.6% with private insurance and 9.5% of the total sample had secondary health insurance (**Table 1**).

**Table 1:** Baseline sociodemographic characteristics of transgender patients seen at a Midwest tertiary medical center.

Characteristic	Total (n=482)	Transgender Female (n=223, 46%)	Transgender Male (n=182, 38%)	Non-binary (n=77, 16%)
Age in years, median (IQR)	24 (21,32)	26 (21,36)	23 (21,28)	27 (22,32)
Race, n (%)				
Black or African American	51 (10.6%)	23 (10.3%)	19 (10.4%)	9 (11.7%)
White	406 (84.2%)	188 (84.3%)	154 (84.6%)	64 (83.1%)
Other	15 (3.1%)	7 (3.1%)	5 (2.7%)	3 (3.9%)
Not disclosed	10 (2.1%)	5 (2.2%)	4 (2.2%)	1 (1.3%)
Ethnicity, n (%)				
Hispanic	12 (2.5%)	5 (2.2%)	4 (2.2%)	3 (3.9%)
Not Hispanic	433 (89.8%)	201 (90.1%)	162 (89%)	70 (90.9%)
Not disclosed	37 (7.7%)	17 (7.6%)	16 (8.8%)	4 (5.2%)
Sex assigned at birth, n (%)				
Male	254 (52.5%)	223 (100%)	0 (0%)	31 (40.3%)
Female	227 (47.3%)	0 (0%)	181 (99.5%)	46 (59.7%)
Intersex	1 (0.2%)	0 (0%)	1 (0.5%)	0 (0%)
Primary Health Insurance, n (%)				
Medicare	17 (3.5%)	13 (5.8%)	4 (2.2%)	0 (0%)
Medicaid				
Illinois	34 (7.1%)	12 (5.4%)	15 (8.2%)	7 (9.1%)
Missouri	35 (7.3%)	20 (9%)	12 (6.6%)	3 (3.9%)
Private Insurance	350 (72.6%)	152 (68.2%)	136 (74.7%)	62 (80.5%)
Self-pay	35 (7.3%)	19 (8.5%)	13 (7.1%)	3 (3.9%)
Other	11 (2.3%)	7 (3.1%)	2 (1.1%)	2 (2.6%)
Secondary Health Insurance, n (%)				
Medicare	3 (0.6%)	3 (1.3%)	0 (0%)	0 (0%)
Medicaid				
Illinois	11 (2.3%)	4 (1.8%)	6 (3.3%)	1 (1.3%)
Missouri	10 (2.1%)	7 (3.1%)	2 (1.1%)	1 (1.3%)
Private Insurance	18 (3.7%)	11 (4.9%)	6 (3.3%)	1 (1.3%)
Other	4 (0.8%)	3 (1.3%)	1 (0.5%)	0 (0%)
None	435 (90.2%)	195 (87.4%)	167 (91.8%)	74 (96.1%)

### Area of deprivation index

**Figure 1:**
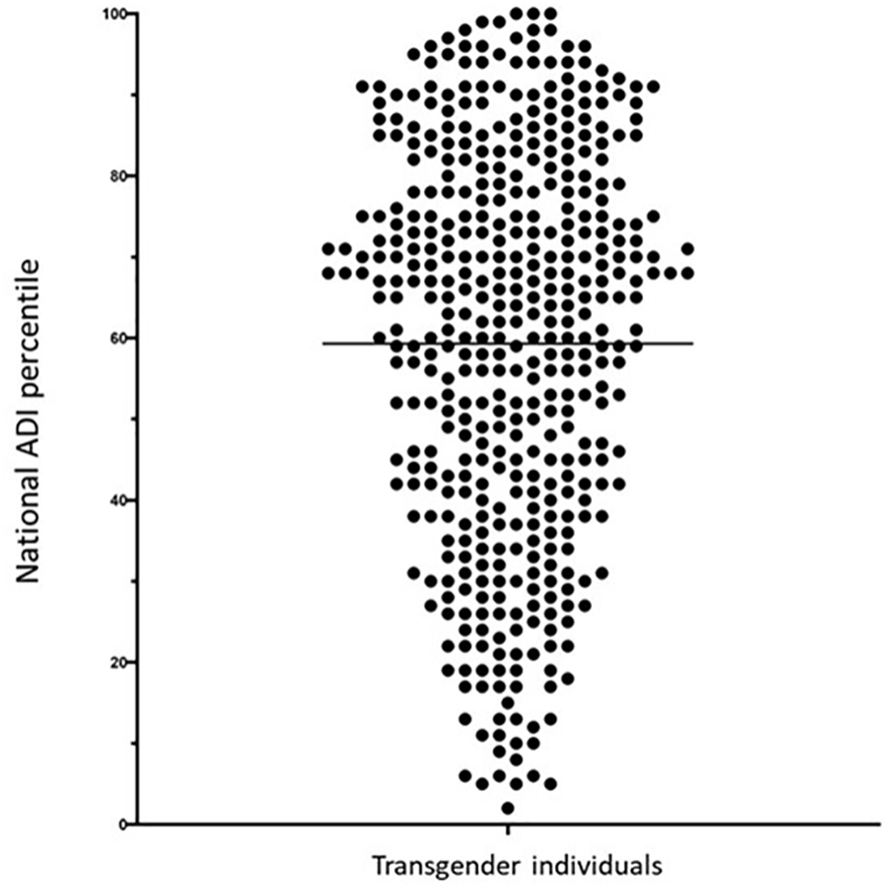
National area of deprivation index percentile of transgender patients seen at an academic center in the Midwest between December 2019 and June 2022. Each circle represents a transgender patient at a national ADI percentile and solid line represent the mean of the sample. The population increases as the national percentile increases to the most disadvantage percentiles.

This study used the national and state ADI. Out of the total sample of 482, we were able to obtain ADI on 451 transgender individuals. The sample studied had a mean national ADI percentile of the 59^th^ percentile. Ten percent of all transgender patients were in the lower quartile of the national ADI (<25^th^ percentile) or least disadvantaged quartile, in comparison to 30% being the top quartile (>75^th^ percentile) or most disadvantaged quartile (**Figure 1**).

**Figure 2:**
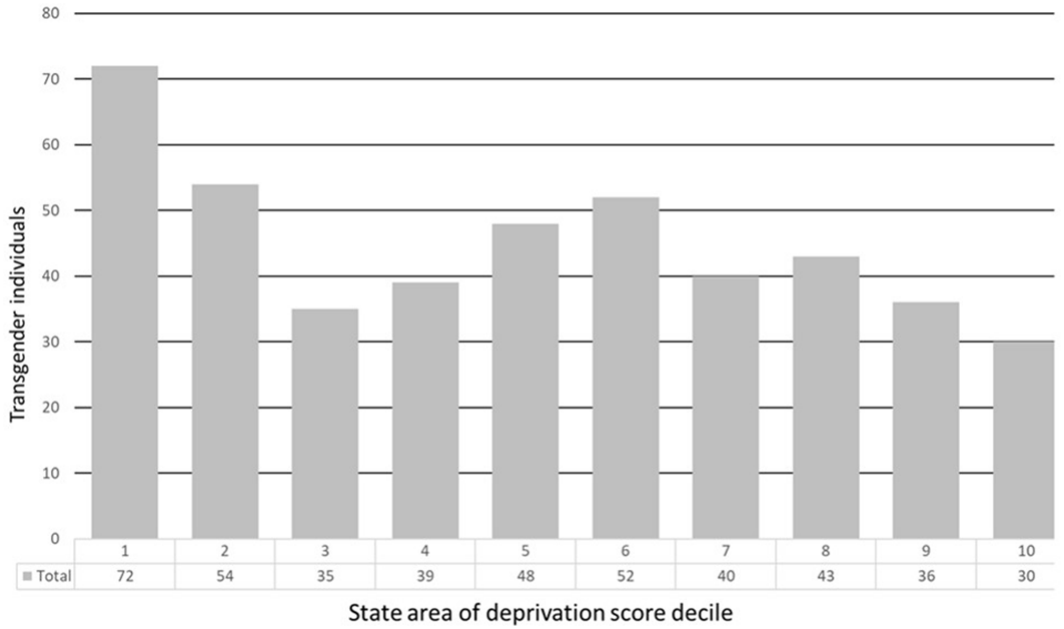
State area of deprivation index decile of transgender patients seen at an academic center in the Midwest between December 2019 and June 2022.

The state ADI is presented in deciles. A decile groups the ADI score into 10 equal sections, following the same interpretation as the national ADI score percentile. In our sample, we obtained a mean state ADI decile of 5 with approximately 16% of patients in the 1^st^ state ADI decile and about 6% in the 10^th^ state ADI decile (**Figure 2**).

### Clinical characteristics

Even though more than 90% of patients had primary insurance, less than half of them had a psychiatrist or therapist and about two thirds reported having a primary care physician, indicating the multidimensional complexity in accessing health care that is not solely dependent in health insurance coverage. Also, 11.6% reported having a history of suicide attempt during their initial evaluation (**Table 2**).

**Table 2:** Baseline self-reported mental health history of transgender patients seen at a Midwest tertiary medical center.

Characteristic (n, %)	Total (n=482)	Transgender Female (n=223)	Transgender Male (n=182)	Non-binary (n=77)
Reports mental health condition	392 (81.3%)	173 (77.6%)	155 (85.2%)	64 (83.1%)
Prior suicide attempt	56 (11.6%)	22 (9.9%)	24 (13.2%)	10 (13%)
Self-harming behavior	21 (4.4%)	5 (2.2%)	13 (7.1%)	3 (3.9%)
Prior psychiatric admission	98 (20.3%)	37 (16.6%)	46 (25.3%)	15 (19.5%)
Reports currently receiving care from…				
Primary care provider	306 (63.5%)	138 (61.9%)	116 (63.7%)	52 (67.5%)
Psychiatrist	109 (22.6%)	42 (18.8%)	46 (25.3%)	21 (27.3%)
Therapist	232 (48.1%)	106 (47.5%)	85 (46.7%)	41 (53.2%)

Eighty percent of the patients reported a mental health diagnosis with the most common diagnoses being depression (n=320, 66.4%), any anxiety disorder (n= 278, 57.7%), and attention deficient hyperactivity disorder ADHD (n= 83, 17.2%) (**Table 3**). Use of psychotropic medications serves as another measure of prevalence of mental health conditions. At their initial patient visit, 48.5% reported a psychotropic medication (**Table 4**). The most common groups of psychotropic medications reported were selective serotonin reuptake inhibitor (SSRI), anxiolytics, and ADHD medications. Among the category other psychotropic medications, the most reported were bupropion (n=22, 4%) and trazadone (n=10, 2%).

**Table 3:** Baseline self-reported mental health conditions of transgender patients seen at a Midwest tertiary medical center.

Conditions (n, %)	Total (n=482)	Transgender Female (n=223)	Transgender Male (n=182)	Non-binary (n=77)
Depression	320 (66.4%)	136 (61%)	130 (71.4%)	54 (70.1%)
Anxiety				
Any anxiety disorder	278 (57.7%)	116 (52%)	117 (64.3%)	45 (58.4%)
Generalized anxiety disorder	40 (8.3%)	13 (6%)	17 (9.3%)	10 (13%)
Agoraphobia	8 (1.7%)	4 (1.8%)	3 (1.6%)	1 (1.3%)
Social anxiety disorder	6 (1.2%)	3 (1.3%)	3 (1.6%)	0 (0%)
Attention Deficit Disorder	83 (17.2%)	44 (19.7%)	25 (13.7%)	14 (18.2%)
Post-traumatic stress disorder	44 (9.1%)	13 (5.8%)	22 (12.1%)	9 (11.7%)
Bipolar disorder	37 (7.7%)	16 (7.2%)	18 (10%)	3 (3.9%)
Obsessive-Compulsive disorder	17 (3.5%)	4 (1.8%)	11 (6%)	2 (2.6%)
Autism Spectrum Disorders	15 (3.1%)	9 (4%)	3 (1.6%)	3 (3.9%)
Eating disorder	12 (2.5%)	2 (1%)	6 (3.3%)	4 (5.2%)
Schizophrenia	10 (2.1%)	5 (2.2%)	5 (2.7%)	0 (0%)
Any personality disorder	10 (2.1%)	2 (1%)	5 (2.7%)	3 (3.9%)
Substance use disorder	9 (1.2%)	5 (2.2%)	3 (1.6%)	1 (1.3%)
Sleep disorder	6 (1.2%)	1 (0.4%)	5 (2.7%)	0 (0%)
Alcoholism	4 (1%)	2 (1%)	2 (1.1%)	0 (0%)
Chronic tic disorder	3 (0.6%)	3 (1.3%)	0 (0%)	0 (0%)
Body dysmorphic disorder	1 (0.2%)	0 (0%)	1 (0.5%)	0 (0%)
Illness anxiety disorder	1 (0.2%)	0 (0%)	1 (0.5%)	0 (0%)
Dissociative identity disorder	1 (0.2%)	0 (0%)	1 (0.5%)	0 (0%)

**Table 4:** Baseline psychotropic medications reported by transgender patients seen at a Midwest tertiary medical center.

Medication	Total (n=482)	Transgender Female (n=223)	Transgender Male (n=182)	Non-binary (n=77)
SSRI (n, %)^1^	130 (27.0%)	50 (22.4%)	53 (29.1%)	27 (35.1%)
SNRI (n,%)^2^	44 (9.1%)	15 (6.7%)	21 (11.5%)	8 (10.4%)
Anxiolytics (n, %)	53 (11%)	17 (7.6%)	26 (14.3%)	10 (13%)
Mood stabilizers (n, %)	12 (2.5%)	4 (1.8%)	6 (3.3%)	2 (2.6%)
Antipsychotics (n,%)	39 (8.1%)	11 (4.9%)	21 (11.5%)	7 (9.1%)
ADHD medications (n,%)	53 (11%)	25 (11.2%)	17 (9.3%)	11 (14.3%)
Tricyclic antidepressants (n,%)	10 (2.1%)	0 (0%)	6 (3.3%)	4 (5.2%)
Other (n,%)	57 (11.8%)	22 (9.9%)	23 (12.6%)	12 (15.6%)
No medication	248 (51.5%)	128 (57.4%)	87 (47.8%)	33 (42.9%)

^1^Selective serotonin reuptake inhibitors.

^2^Serotonin and norepinephrine reuptake inhibitors.

After their initial evaluation, the GAHT regimen most commonly prescribed to transgender females was sublingual 17-beta estradiol co-prescribed with spironolactone as an androgen blocker. The GAHT regimen most commonly prescribed to transgender males was intramuscular or subcutaneous testosterone (**Table 5**).

**Table 5:** Gender affirming hormone therapy prescribed to transgender patients seen at a Midwest tertiary medical center during their initial visit.

Medication	Total (n=482)	Transgender Female (n=223)	Transgender Male (n=182)	Non-binary (n=77)
Estradiol (n, %)	236 (49%)	211 (94.6%)	0 (0%)	25 (32.5%)
Oral	6 (1.3%)	6 (2.7%)	0 (0%)	0 (0%)
Sublingual	180 (37.3%)	160 (71.7%)	0 (0%)	20 (26%)
Intramuscular/Subcutaneous	20 (4.1%)	18 (8.1%)	0 (0%)	2 (2.5%)
Transdermal	30 (16.1%)	27 (12.1%)	0 (0%)	3 (3.8%)
Testosterone Blocker/Suppression	184 (38.2%)	169 (75.8%)	0 (0%)	15 (19.5%)
Spironolactone	171 (35.5%)	158 (70.9%)	0 (0%)	13 (16.9%)
Bicalutamide	11 (2 %)	9 (4%)	0 (0%)	2 (2.5)
Other	1 (0.2%)	1 (0.4%)	0 (0%)	0 (0%)
GnRH agonist	1 (0.2%)	1(0.4%)	0 (0%)	0 (0%)
Progesterone (n, %)	60 (12.4%)	53 (23.7%)	0 (0%)	7 (9.1%)
Testosterone (n, %)	210 (43.6%)	1 (0.4%)	173 (95.1%)	36 (49.4%)
Intramuscular/Subcutaneous	179 (85.2%)	1 (0.4%)	150 (82.4%)	28 (36.4%)
Transdermal patches	6 (2.9%)	0 (0%)	5 (2.7%)	1 (1.3%)
Transdermal gel	25 (11.9%)	0 (0%)	18 (9.9%)	7 (9.1%)
No medication	0 (0%)	0 (0%)	9 (5%)	0 (0%)

### Effect of medical and sociodemographic factors on self-reported mental health diagnosis

A multivariable logistic regression model was constructed to assess associations between self-reported mental health diagnosis and medical and sociodemographic factors including gender identity, primary health insurance, initiation or continuation of GAHT, referral from the Pediatric Center, and the Area of Deprivation Index corresponding to the individual’s zip code or residence at a state and national level. The covariates that were included in the model were age and race. Logistic regression results (**Table 6**) show none of the factors was significantly associated with self-reported mental health diagnosis during the initial visit.

**Table 6:** Effect of factors on mental health diagnosis after adjusting for the effect of age and race in transgender patients seen at a Midwest tertiary medical center.

Factors	Mental Health Diagnosis		Odds Ratio (95% CI)	p-value
	No (n=86)	Yes (n=386)		
Gender identity				0.3601*
Transwoman	47 (54.7%)	171 (44.3%)	Reference	
Transman	26 (30.2%)	152 (39.4%)	1.48 (0.85, 2.56)	0.1644
Nonbinary	13 (15.1%)	63 (16.3%)	1.3 (0.65, 2.62)	0.4571
Primary health insurance:				0.7304*
Medicare	3 (3.5%)	13 (3.4%)	2.57 (0.58, 11.33)	0.2130
IL Medicaid	5 (5.8%)	29 (7.5%)	1.81 (0.64, 5.17)	0.2662
MO Medicaid	7 (8.1%)	28 (7.3%)	1.12 (0.45, 2.8)	0.8088
Private insurance	65 (75.6%)	278 (72%)	Reference	
Self-pay	6 (7%)	27 (7%)	1.31 (0.49, 3.48)	0.5927
Other	0 (0%)	11 (2.8%)	NA	0.9847
New to GAHT				
Yes	42 (49.4%)	212 (55.4%)	1.21 (0.73, 2)	0.4622
No	43 (50.6%)	171 (44.6%)	Reference	
Referral From Pediatric Center				
Yes	5 (5.6%)	24 (6.3%)	0.86 (0.31, 2.42)	0.7782
No	80 (93%)	359 (93%)	Reference	
ADI - State	4.7 ± 2.9, n=78	5 ± 2.9, n=357	1.06 (0.97, 1.17)**	0.1778
ADI national	57.4 ± 25.4, n=78	59.5 ± 24, n=357	1.01 (1, 1.02)**	0.2094

n, sample size,

When less than the entire cohort provided data, sample size (n) or the denominator is also reported.

*P-value associated with type 3 p-value from logistic regression model tests the overall effect of factor on outcome.

**The Odds Ratio is expressed for 1 unit increase for each continuous factor.

### Effect of medical and sociodemographic factors on having a mental health care provider

A multivariable logistic regression model was constructed to assess associations between having a mental health care provider with medical and sociodemographic factors including gender identity, primary health insurance, initiation, or continuation of GAHT, referral from the Pediatric Center, and the Area of Deprivation Index corresponding to the individual’s zip code of residence at a state and national level. The covariates that were included in the model were age and race. Logistic regression results (**Table 7**) show patients who were new to GAHT were more likely to report having a mental health care provider in the adjusted model (aOR 2.0, 95% CI 1.4-2.9).

**Table 7:** Effect of factors on having a mental health care provider in transgender patients seen at a Midwest tertiary medical center.

Factors	Mental health care provider		Odds Ratio (95% CI)	p-value
	No (n=241)	Yes (n=231)		
Gender identity				0.6111*
Transwoman	117 (48.5%)	106 (45.9%)	Reference	
Transman	97 (40.2%)	85 (36.8%)	0.97 (0.65, 1.43)	0.8680
Non-binary	36 (14.9%)	41 (17.7%)	1.26 (0.75, 2.11)	0.3876
Primary health insurance:				0.2562*
Medicare	11 (4.7%)	6 (2.6%)	0.53 (0.19, 1.46)	0.2169
IL Medicaid	18 (7.5%)	16 (6.9%)	0.86 (0.42, 1.74)	0.6726
MO Medicaid	18 (7.5%)	17 (7.4%)	0.91 (0.46, 1.83)	0.7965
Private insurance	172 (71.3%)	178 (77.1%)	Reference	
Self-pay	23 (9.5%)	12 (5.2%)	0.5 (0.24, 1.04)	0.0655
Other	8 (3.3%)	3 (1.3%)	0.36 (0.09, 1.39)	0.1389
New to GAHT	n=247	n=231		
Yes (treatment-naïve)	114 (46.2%)	145 (62.8%)	1.97 (1.36, 2.84)	0.0003
No (continuation of therapy)	133 (53.8%)	86 (37.2%)	Reference	
Referral From Pediatric Center	n=247	n=231		
Yes	20 (8.1%)	9 (3.9%)	0.46 (0.21, 1.03)	0.0598
No	227 (91.9%)	222 (96.1%)	Reference	
ADI - State	5.1 ± 3, n=231	4.9 ± 2.8, n=213	0.97 (0.91, 1.04)*	0.4294
ADI national	59.9 ± 24.6, n=231	58.6 ± 23.8, n=213	1 (0.99, 1.01)*	0.5859

n, sample size,

When less than the entire cohort provided data, sample size (n) or the denominator is also reported.

*P-value associated with type 3 p-value from logistic regression model tests the overall effect of factor on outcome.

**The Odds Ratio is expressed for 1 unit increase for each continuous factor.

## Discussion

Research in transgender adults has been recognized as an understudied area, hence, the importance of this study characterizing sociodemographic factors and mental health diagnosis in transgender adults seen at a Midwest academic center. It also provides a unique perspective on the impact of sociodemographic factors in history of mental health diagnosis and access to a mental health provider. Among the sociodemographic factors, we included the state and national area of deprivation index to provide a consistent and standardized measure that might be difficult to capture in clinical settings (24).

Our study found transgender individuals seen in our clinic have a 81% life-time prevalence of mental health conditions with depression and anxiety being the most common, with a life-time self-reported prevalence of 66.4% and 57.7% respectively. Our results are similar to those described in a US cross-sectional study of 25,233 transgender inpatient encounters (10), which showed that 76.7% of all transgender inpatient encounters had at least one mental health disorder diagnosis, and these hospital encounters were more than 3 times as likely to have a mood disorder diagnosis than cisgender inpatient encounters. These results extend observations from the multicenter European Network for the Investigation of Gender Incongruence study showing that 70% of transgender participants have a life-time prevalence of affective and anxiety disorder (26, 27). For contrast, the lifetime prevalence of depression and anxiety in US adults is 47.6% and 31.1%, respectively (28).

Furthermore, there were no differences in the life-time prevalence of any mental health condition between gender identities nor a relationship between sociodemographic factors and reported history of mental health diagnosis. Prior studies have shown transgender individuals have higher rates of mental health conditions compared to their cisgender peers (29, 30). These findings have been explained by the minority stress model, which refers to the psychosocial stressors that transgender individuals experience on account of their (gender) minority status (31, 32). After adjusting for socio-economic factors, our study findings support the conclusions of prior research that this difference in mental health diagnosis prevalence between transgender and cisgender individuals is, in part, due to gender dysphoria, minority stress and its implications as a proximal stressor (32, 33). Socioeconomic factors were not associated with the prevalence of mental health diagnosis in our sample, while socioeconomic status has been implicated as an important moderator of stress and psychological health outcomes in other populations (34–36) Transgender individuals experience employment and education discrimination in our society which impact socioeconomic status, potentially confounding the relationship between mental health and socioeconomic status in this population.

A growing body of literature shows positive benefits of GAHT on mental health, with the most consistent effects being decreases in depressive symptoms, psychological distress, and potentially reduced general anxiety (37, 38), which could explain why patients who are seen for consultation for initiating GAHT are almost two times more likely to have seen their mental health provider over the last year than those seen for continuation of therapy, and the reason why patients referred for continuation of therapy may be less likely to need ongoing mental health support. In our study population, approximately one in every 8 transgender adults reported a prior suicide attempt during their initial appointment, which is approximately 5 times higher than the lifetime prevalence of suicidal attempts in the general population based on previously-reported data in a US cross-national sample (39).

This study has several important strengths including the large sample size and the description of binary and non-binary gender identities with subsequent analysis for potential differences between the groups. Also, the collection of data included zip codes, allowing the ADI to be utilized as a standardized method for incorporating socioeconomic data (that would be otherwise difficult to obtain from medical records) into our analysis. The study also has some limitations. First, this was a retrospective chart review. Data collection was limited to information that was included in the electronic health record for clinical rather than research purposes. Hence, it is possible that information about visits to mental health care providers and mental health diagnoses were incompletely captured. Information on mental diagnoses was self-reported in many cases and could be partial or limited to the recall of patients during appointments. ADI data capture population level socioeconomic status characteristics and are used as a proxy for individual-level social determinants of health. We recognize that population-level data have limits in this regard and may incompletely represent individual circumstances and needs. Additionally, as a cross-sectional analysis, we can only establish relationships of association and not causal effect between the factors and outcomes studied.

## Conclusions

In summary, this study provides insight on the psychosocial characteristics of transgender adults during their initial appointment at a Midwest transgender center. It demonstrates that transgender adults continuing GAHT are less likely to have seen a mental health provider in the prior year than they were before initiating GAHT. We show a lack of relationship between sociodemographic factors and the prevalence of mental health conditions in our study population.

## Data Availability

The original contributions presented in the study are included in the article/supplementary material. Further inquiries can be directed to the corresponding author.
